# External load of the tasks planned by teachers for learning handball

**DOI:** 10.1371/journal.pone.0265745

**Published:** 2022-04-05

**Authors:** Sebastián Feu, Javier García-Rubio, Sergio J. Ibáñez, Antonio Antúnez

**Affiliations:** 1 Training Optimization and Sports Performance Research Group (GOERD), University of Extremadura, Caceres, Spain; 2 Faculty of Education, University of Extremadura, Badajoz, Spain; 3 Faculty of Sport Science, University of Extremadura, Caceres, Spain; Qatar University College of Education, QATAR

## Abstract

The load in tasks planned for sports teaching in physical education classes has received little attention. The purpose of this study was therefore to analyze the external load, eTL, in the tasks designed by physical education teachers from the in-service and pre-service stages, for teaching handball in primary education, and to compare them with the tasks included in the lesson plans designed for handball using the tactical games teaching model. An associative, comparative and cross-sectional methodology was used. Twenty-three teachers, five in the in-service phase and eighteen in the pre-service phase, designed lesson plans for teaching handball, which were compared with lesson plans validated by a panel of experts. The analysis was performed on 1,232 tasks or analysis units. eTL was categorized using the Integrated analysis system of training tasks (SIATE) instrument. A descriptive and associative analysis was made of the variables that make up the eTL and an inferential analysis of the eTL using non-parametric tests. The total eTL of the tasks planned by the in-service and pre-service teachers was low, and significantly lower than the tasks planned using the tactical games model, which showed a high level.

## Introduction

Sports are one of the most commonly used contents by physical education (PE) teachers in their curricular designs [1, 2]. Also, there is concern in PE sports to adapt the demands to contribute to their students’ development. For this reason, it is necessary to know the physical demands elicited by the learning tasks to adjust the load supported by the students [3, 4]. The quantification of the load, the sum of the stimuli received by the student’s organism, is a key element for being able to analyze these demands [5]. The scientific literature includes studies focused on analyzing the nature of the effort, the internal load (*internal Training Load*, *iTL*) and the external load (*external Training Load*, *eTL*) in training and competition [6–8], and even in PE classes [9, 10], although the latter are scarce.

Training load can be quantified objectively and subjectively [11]. External load is the result of all the efforts that the players have made during a task [12], in terms of distance, velocity and acceleration. eTL can be measured, quantified and monitored objectively using inertial devices [13, 14]. Among the objective variables, player load is an indicator that is obtained from the accelerations produced in the three planes of body movement [15]. These methods are expensive regarding the equipment used and interfere with the development of a physical education class. As an alternative to the expensive instruments needed to monitor eTL, others have arisen with an observational character, through which eTL can be calculated subjectively. The Integrated analysis system of training tasks (SIATE) instrument [16], proposes the quantification of eTL with the recording of six subjective variables. The quantification of eTL with the SIATE presents a high correlation with eTL (Player load) and iTL (HR) obtained with inertial measurements units (IMUs) [17, 18].

Adequate planning of the teaching-learning process is necessary to identify the requirements of the tasks used in the classroom [19]. Teachers should avoid improvisation and lack of knowledge about the demands of the tasks for the students. The task load is conditioned by the teaching method, the organization of the lesson [10, 12, 19, 20], and the teacher’s characteristics. Pre-service teachers plan tasks with a low eTL, which is related to the teaching model used [21–23].

The methodologies used to teach invasion sports can have repercussions on the task load [4]. Sports teaching has basically followed two methodological approaches, the Student-Centered approach such as the tactical game approach and the Teacher-Centered approach such as direct instruction [24]. eTL is higher in teaching units (TU) planned according to the tactical games approach model compared with the Teacher-Centered approach, both in soccer [19] and in basketball [20]. These differences have been verified in practice using the objective (Player Load) [10] and subjective (SIATE) observational systems [12].

It is important to study the load and intensity provoked by the tasks to know if they are significant for the development of the students’ fitness and health. The WHO recommends that children and adolescents should devote an average of 60 minutes a day to moderate physical activity that is mainly aerobic and at least three days a week to intense aerobic activities [25]. These demands should be covered by their physical education classes [4]. Some studies report experiences of low physiological intensity in physical education sessions devoted to sports teaching [26].

The intensity of tasks can vary according to the pedagogical model selected by the teacher, the learning media and the game situations [10, 26, 27]. Some investigations present the limitation that it is not known if the lesson plans used according to a pedagogical model have been validated by a panel of experts [28], thus hindering comparisons among programs. In this study the teachers’ tasks have been compared with those of a lesson plan designed according to the tactical games approach and validated by a panel of experts.

To the best of our knowledge, studies analyzing eTL in the school context are scarce and to date there are none that investigate the planning of external load according to the type of teacher, whether in-service or pre-service. It has been hypothesized that: i) Pre-service and in-service teachers plan learning tasks with a low external load; ii) Tasks with a low external load are associated with unopposed learning situations and exercise-based learning media, while higher loads are associated with situations played with opposition; iii) Pre-service and in-service teachers plan the load of the tasks to be lower than those of a lesson plan designed under a tactical games model. Thus, the main purpose of this study was: i) to analyze eTL in tasks designed by in-service and pre-service teachers for teaching handball in primary education; and ii) to compare the tasks designed by the teachers with a lesson plan designed and validated under the tactical games approach (TGA) model.

## Materials and methods

### Design

An associative, comparative cross-sectional design was used [29], aimed at discovering the external load of the tasks and the type of task used by different groups of teachers.

### Sample

A total of 1,232 tasks were analyzed from teaching units devoted to the teaching of handball in schools, of which 239 cases corresponded to the tasks planned by six in-service teachers, 926 corresponded to the lesson plans designed by eight pre-service teachers and 67 tasks were from a teaching unit planned using the tactical games model according to the SCA methodology.

### Variables

Two independent variables were used: i) The type of teacher who designed the lesson plan: pre-service teacher or in-service teacher and unit planned by experts using a small-sided model; ii) The lesson structure: warm-up (n = 320), main activity (n = 525) and cool down (n = 177).

eTL was calculated using the variables proposed in the Integrated analysis system of training tasks (SIATE) [16]. This system incorporates six primary variables to characterize eTL: *Level of Opposition*, *Task Density/Intensity*, *Number of Players*, *Competitive Load*, *Game Area*, and *Cognitive Involvement*. Each eTL variable is structured as an ordinal categorical system with 5 levels. Using the primary variables that determine the eTL, the teacher can subjectively quantify the load provoked by the tasks thus obtaining a secondary variable: external task load. The value of this variable oscillates between 6 and 30, with four ranges to categorize the level: 6–12 (very low), >12–18 (low), >18–24 (high) and >24–30 (very high) [16].

The tasks were contextualized using the SIATE pedagogical variables [16]: Teaching means and Game situations.

#### Instruments

Coding of the eTL and pedagogical variables was performed using the SIATE instrument [16], which has been used to calculate eTL in numerous studies [12, 19–21].

The game situations were classified into five groups: Without opposition (WO); Individual Game (IG) (1x1); Numerical inequality SSG (SSG-I) (2x1, 3x2, 4x2…); Numerical equality SSG (SSG-E) (2x2, 3x3, 4x4); and Full game (FG) (5x5, 6x6, 7x7). The learning media were categorized into four groups: Exercises; Specific game; Non-specific game; and Sport / Pre-sport / Adapted sport, Table 1.

**Table 1:** Synthesis of the eTL and pedagogical variables.

eTL Variables		Description
Degree of opposition	DO	Degree of opposition based on the number of opponents in the task.
Density of the task	DT	Indicates subjectively the intensity with which the task is developed.
Percentage of simultaneous performers	PSP	Indicates the level of participation of the players during the task.
Competitive load	CL	Refers to the emotional and psychological load that the players support when they have to carry out a task under pressure to achieve a result.
Game space	GS	The space in which the players have to carry out the proposed tasks.
Cognitive implication	CI	Refers to the tactical load, i.e., the attention that the player has to give to team mates and opponents.
eTL task load	eTL	Obtained by adding the value assigned to each of the six eTL variables (1 to 5 points). DO + DT + PSP + CL + GS + CI = quantification of eTL.
Pedagogical Variables		Description
Game Situations	GS	Groups of players that the teachers and coaches design for each of the tasks (e.g., 2 × 1; 2 being the number of attackers and 1 the number of defenders).
Teaching means	TM	Sports motor activities that serve to develop technical and tactical contents.

### Procedure

The study was structured in several phases: i) Selection and design of the instrument to collect the data. The SIATE instrument was selected [16].

ii) Design and validation of a TU centered on the tactical games model for the teaching of handball (TG-H). First, a TU was designed based on the literature about the teaching of invasion sports using a SCA and the tactical games model [30–32], which was validated by a panel of experts. The experts had a PhD. in Physical Activity and Sports Sciences, with the highest qualification in an invasion sport and publications in scientific journals on this topic.

iii) Collection of the teaching units. The in-service and pre-service teachers were asked for the lesson plans they had designed for the initiation in handball of fifth- and sixth-year students of primary education. The teachers were informed that their participation was voluntary and anonymous and the data provided would be protected by the Spanish law on data protection. Approval was requested for this study from the University Bioethics Committee (Ref. 247/2019).

In Spain, primary school teachers have to pass an educational process at the university with initial training lasting four years. After three years of training as a generalist teacher, the students have a fourth specific year in physical education training, along with an internship period in schools. The group of teachers in the pre-service phase, who were Elementary Education degree students in their fourth year, were asked to design a teaching unit with handball as its content. The group of in-service teachers were active teachers in Spanish public educational schools. They were asked for their plans for teaching handball in primary education.

The inclusion criteria for the teachers who provided the lesson plans analyzed in the study were: i) To be working as a physical education teacher in primary education at the moment of conducting the study and to have more than ten years’ experience; and ii) for the pre-service teachers, they had to be in an internship as a physical education teacher in a primary school. Both groups of teachers had to design and apply a lesson plan for teaching the sport of handball with a minimum of six sessions.

iv) Training of the coders: The following phases were implemented [33]: Theoretical, Practical and Training. Once the Training phase had finished intra-observer reliability was calculated using Cohen’s Kappa, for which 10% of the tasks in the warm-up and main activity phase were coded (n = 84 coded units) 15 days apart. Almost perfect reliability was obtained for the variables Game situation (*k* = 0.88) and Degree of opposition (*k* = 0.83), and substantial reliability for the variables Density of the task (*k* = 0.78) Game space (*k* = 0.75), Percentage of simultaneous participants (*k* = 0.70), Competitive load (*k* = 0.74) and Cognitive implication (*k* = 0.70). Interobserver agreement in all the variables was perfect according to (Landis & Koch, 1977; Viera & Garrett, 2005) [34, 35].

v) Coding of the sample: the sample was coded using the record sheet created with the selected variables, for the subsequent analysis of the data.

### Statistical analysis

A descriptive analysis was performed of all the study qualitative (*n* and *%*) and quantitative (*n* and *%*) variables. Association between the categorical variables was estimated using the Chi square (*χ*^*2*^) and Cramer’s V (*V*_*c*_) coefficients [36]. It was necessary to perform the Fisher’s exact test (f) with the Monte Carlo method for some categorical variables where the frequencies were very low [37]. The interpretation of the degree of association between the variables was performed using Adjusted Standardized Residuals (*ASR*) (>|1.96|) from the contingency tables [37]. The degree of association between variables was estimated using four ranges [38]: Small (< .100), Low (.100-.299), Moderate (.300-.499) and High (>.500). A figure was drawn depicting a correspondence analysis to characterize the type of task used by the teachers and the TG-H model.

Differences in eTL were analyzed according to the type of teacher using the Kruskal Wallis H test. The comparison between groups was performed with the Mann Whitney U test applying the Bonferroni correction. Effect size was calculated using Cohen’s d, following the ranges established by [39]: < .000 (adverse), .000 - .199 (no effect), .200 - .499 (small), .500 - .799 (intermediate) and .800 - ≥ 1.000 (large). The statistical software used was IBM SPSS version 21 (IBM Inc., Chicago, IL, USA). Statistical significance was set at *p* < 0.05.

## Results

Table 2 shows the descriptive results and the ASR of the categories of quantification of the external load for each type of teacher and the TG-H model. Cramer’s V coefficient shows associations with a low effect size in most of the variables, except the variable competitive load which was moderate. However, the ASR indicate that there are more cases than expected in the highest category of each variable in the TG-H teaching model (ASR between 2.0 and 9.8), except in game space. The in-service teachers, in more cases than expected, used repeated movements in large game spaces (*ASR* = 5.7) and competitive load in matches of all types (*ASR* = 6.1).

**Table 2:** Descriptive analysis of eTL variables.

		In-service			Pre-service			TG-H				
*Variable*	*Category*	*n*	*%*	*ASR*	*n*	*%*	*ASR*	*n*	*%*	*ASR*	*Vc*	*p*
DO	Without opposition	139	58.2	3.3*	447	48.3	-.3	12	17.9	-5.2*	.144	.000
	3 more students	32	13.4	-.3	133	14.4	.6	8	11.9	-.5		
	2 more students	0	0	-1.6	11	1.2	1.9	0	0	-.8		
	1 more student	13	5.4	.6	37	4.0	-2.1*	8	11.9	2.9*		
	Numerical equality	55	23.0	-3.3*	298	32.2	.5	39	57.4	4.8*		
DT	Walking	63	26.4	-.3	257	27.8	.9	14	20.9	-1.2	.259	.000
	Slow pace	61	25.5	3.1*	168	18.1	.7	0	0	-4.0*		
	Intensity with rest	81	33.9	1.2	266	28.7	-2.5*	30	44.8	2.6*		
	Intensity without rest	5	2.1	-7.1	210	22.7	7.8*	4	6,0	-2.6		
	High intensity without rest	29	12.1	4.5*	25	2.7	-8.3*	19	28,4	8,0*		
PSP	<20%	22	9.2	-4.2*	209	22.6	6.0*	0	0	-4.0*	.150	.000
	21–40%	9	3.8	1.1	25	2.7	-.2	0	0	-1.4		
	41–60%	17	7.1	2.8*	31	3.3	-2.0*	1	1,5	-1.1		
	61–80%	8	3.3	2.0*	11	1.2	-2.8*	3	4.5	1.7		
	>81%	183	76.6	1.5	650	70.2	-3.5*	63	94.0	4.0*		
CL	Activity without competition	115	48.1	8.9*	187	20.2	-7.4*	12	17.9	-1.5	.309	.000
	Activity with technical skills	21	8.8	-6.6*	293	31.6	8.6*	0	0	-4,9*		
	Opposition without scoring	55	23.0	-.7	235	25.4	.8	16	23,9	-.2		
	Opposition with scoring	15	6.3	-5.4*	191	20.6	3.2*	23	34.3	3.4*		
	Matches of all types	33	13.8	6.1*	20	2.2	-9.1*	16	23.9	6.7*		
GS	Static activity	37	15.5	-.9	166	17.9	.8	12	17.9	.1	.197	.000
	Small spaces	45	18.8	2.0*	125	13.5	-2.1*	11	16.4	.4		
	Intermediate spaces	99	41.4	5.1*	234	25.3	-3.9*	14	20.9	-1.4		
	Large spaces	38	15.9	-7.5*	390	42.1	6.7*	26	38.8	.3		
	Repeated movements in large spaces	20	8.4	5.7*	11	1.2	-6.1*	4	6.0	1.6		
CI	Individual intervention	82	34.3	3	246	26.6	-1.0	0	0	-5.1*	.184	.000
	Intervention of 2 students	62	25.9	-3.2*	340	36.7	2,5*	26	38.8	.7		
	Intervention of 3 students	12	5.0	-1.6	70	7.6	0	11	16.4	2.8*		
	Intervention of 4 students	28	11.7	6.3*	21	2.3	-6.1*	4	6.0	.7		
	Intervention of two teams	55	23.0	-1.5	249	26.9	.1	26	38.8	2.3*		

*Adjusted Standardized Residual, *ASR >* |1.96|.

**Fig 1:**
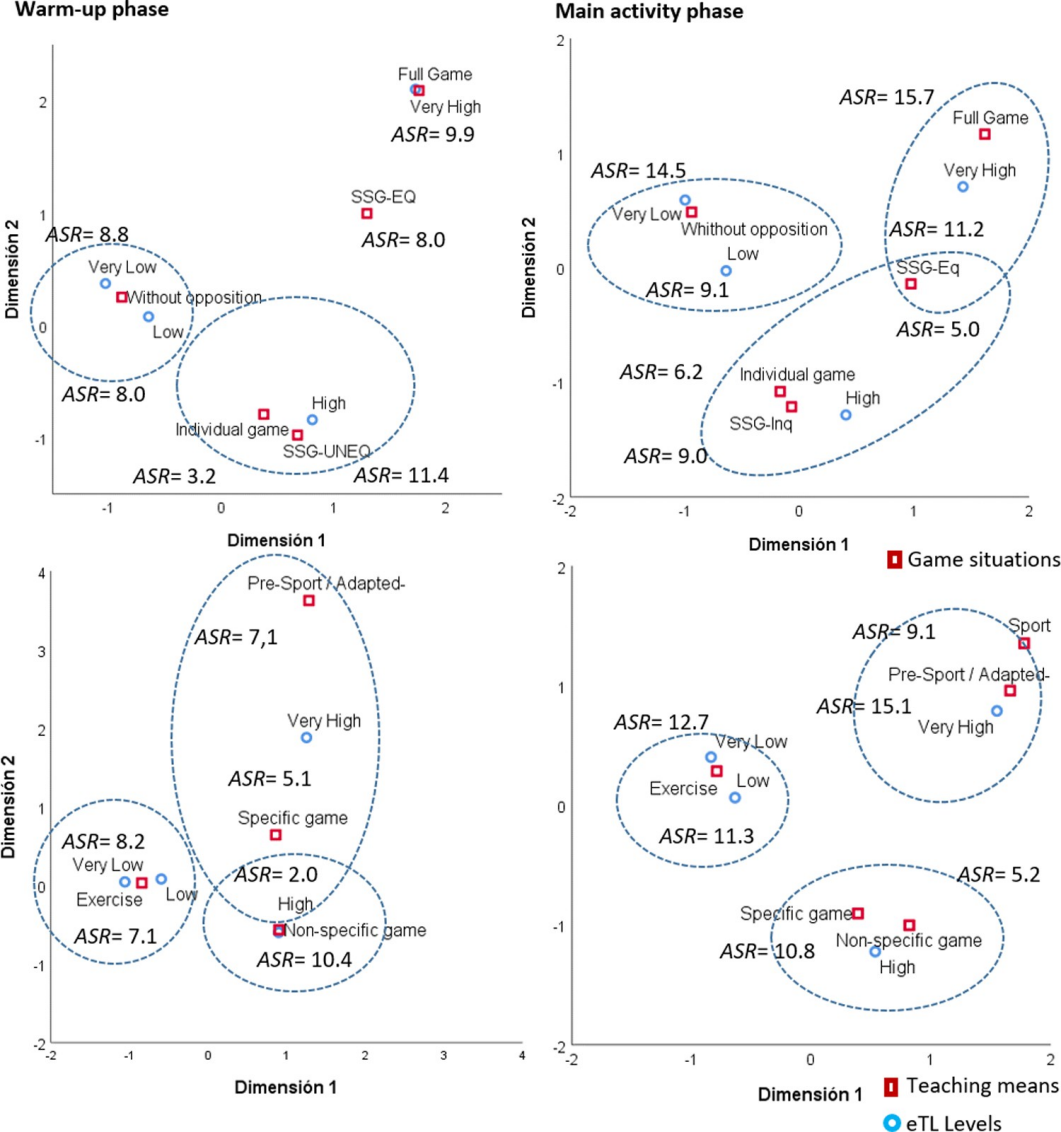
Associations between means and learning situations and the levels of eTL.

Fig 1 presents the associations between the means and learning situations with the eTL levels categorized in four groups, for the warm-up and main activity, using correspondence analysis and the *ASRs*. There are more cases than expected in the exercises and situations without opposition that are associated with a low or very low eTL. In the main activity phase, the task with a specific game (*ASR* = 10.8) is associated with a high eTL, and the tasks Sport (*ASR* = 9.1) and Pre-Sport (*ASR* = 15.1) are associated with a very high eTL. The tasks planned under the small groups format (SSG-Eq) present a high eTL (*ASR* = 8 in the warm-up phase and *ASR* = 5 in the main activity phase). The full game is associated, with more cases than expected (*ASR* = 9.9 in the warm-up phase and *ASR* = 15.7 in the main activity phase), with a very high external load.

**Fig 2:**
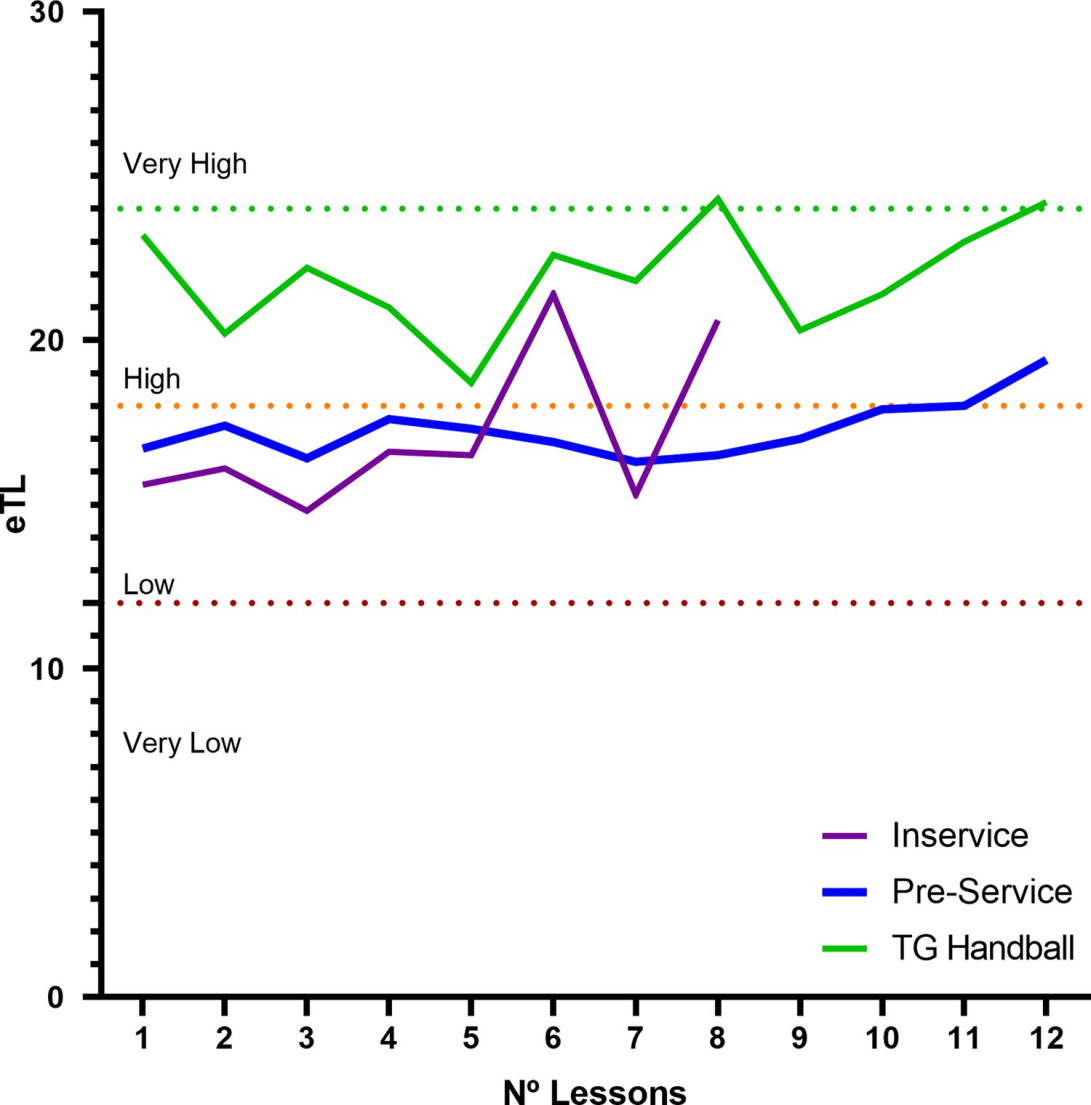
eTL per session for the types of teachers. The in-service teachers planned ≤ 8 Lessons.

Fig 2 shows that the in-service teachers design the TU with fewer sessions than the pre-service teachers and the TG-H program. A large proportion of the tasks designed by the in-service and pre-service teachers are below the high level. The TU designed using the TG-H model is at the high level.

The descriptive statistics of the eTL indicate that the highest scores were achieved with the TG-H model, Table 3. The distribution of the data did not satisfy the assumption of normality (*p*>.05). The Kruskal Wallis H test indicated that there were differences in the planning of the external load according to the type of teacher (*X*^*2*^ = 37.58; *gl* = 2; *p* < .001).

**Table 3:** eTL variables according to the type of teacher.

	In-service				Pre-service				TG-H				
	*X*	*SD*.	*As*.	*Kur*.	*X*	*SD*.	*As*.	*Kur*.	*X*	*SD*.	*As*.	*Kur*.	*K-S*
eTL	16.79	6.30	.69	-.67	17.20	5.64	.35	-.87	21.84	6.14	-.55	-.57	3.80*

*p < .05.

The comparative analysis among the groups showed significant differences in eTL according to the type of teacher, with differences between the TG-H unit and that of the in-service teachers (*U* = 4591.50; *p* < .001; *d* = .641) and the pre-service teachers compared to the other two groups (*U* = 17731,50; *p* < .000; *d* = 1.45), Table 4.

**Table 4:** Results of the relation and degree of association between the eTL variables.

*Variable*	*Teacher*	*Ranks*	*X* ^*2*^	*P*	*Groups Ranks*	*U*	*p*	*d*
eTL	In-service	568.67	37.58	.000	In-service vs. TG-H 139.21–204.47	4591.50	.000*	.641
	Pre-service	610.81			In-service vs. Pre-service 549.46–591.66	102641.00	.083	.144
	TG-H	865.82			Pre-service vs. TG-H 482.65–695.35	17731.50	.000*	1.45

*p < .05.

**Fig 3:**
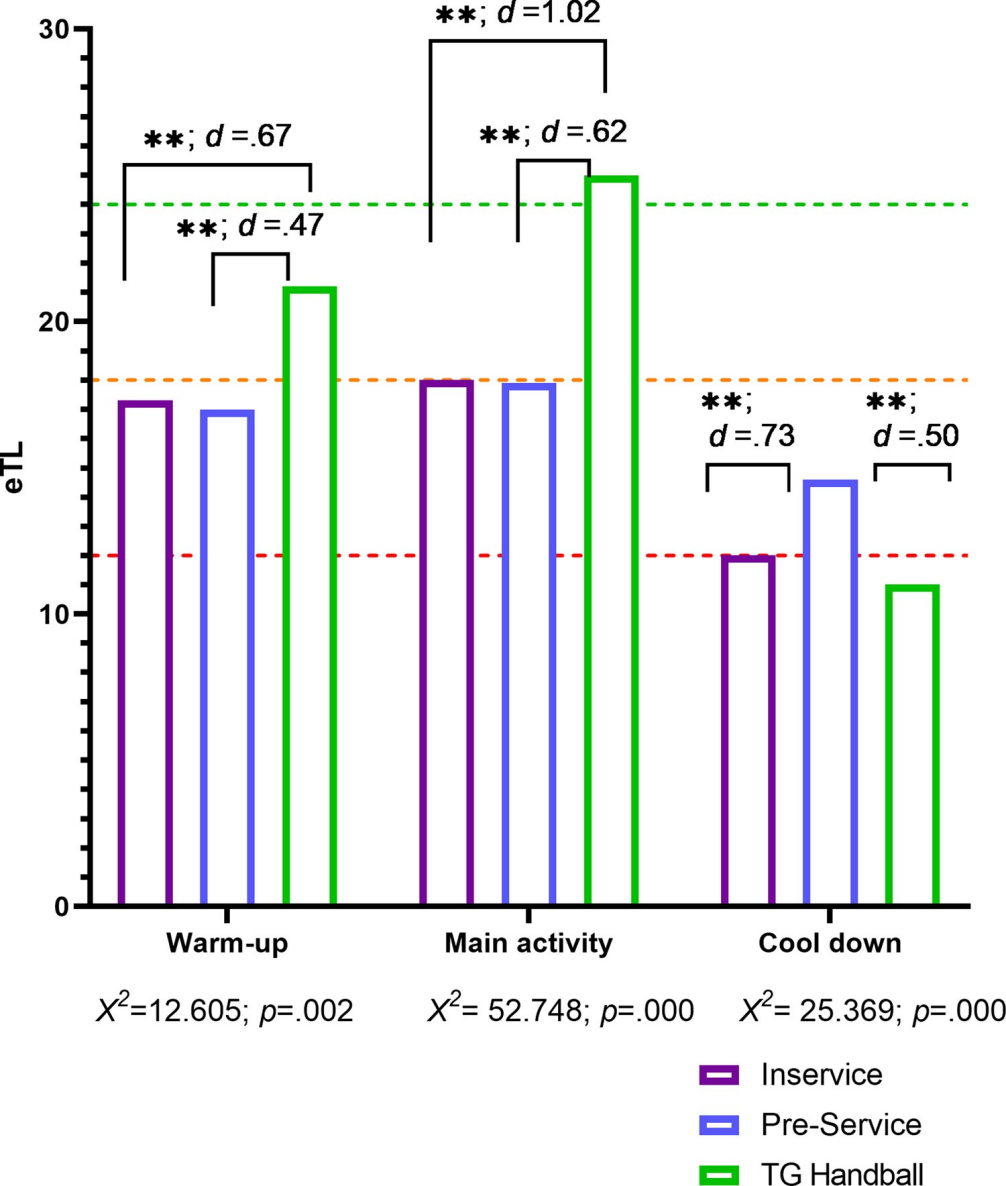
Differences in eTL according to the type of teacher in the different parts of the session (** *p* < .01).

The planning of external load is gradual and differentiates the parts of the session, showing significant differences according to the type of teacher. The learning unit designed following the TG model showed the highest means for the warm-up and main activity (Fig 3). Significant differences were revealed in the three parts of the session regarding the eTL planned by the teachers and experts (*p* < .01).

In the warm-up significant differences were observed between the TG-H model and the two groups of teachers, in-service PE teachers (*U* = 197.50; *p* < .01; *d* = .675) and pre-service PE teachers (*U* = 547.50; *p* < .001; *d* = .468). In the main activity phase differences were found between the in-service (*U* = 1099.00; *p* < .001; *d* = 1.02) and pre-service teachers (*U* = 3984.00; *p* < .001; *d* = .624) and the TG-H unit. In the cool down phase significant differences were found between all the groups compared, Table 5.

**Table 5:** Inferential analysis of eTL according to the teacher.

*Variable*	*Teacher*	*Average range*	*X* ^*2*^	*p*	*Groups Average range*	*U*	*p*	*d*
Warm-up	In-service (n = 69)	161.59	12.605	.002	In-service vs. TG-H 37.86 / 59.04	197.50	.004*	.675
	Pre-service (n = 239)	155.57			In-service vs. Pre-service 158.72 / 153.28	7954.00	.654	.051
	TG-H (n = 12)	252.42			Pre-service–TG-H 122.29 / 199.88	547.500	.000*	.468
	In-service (n = 131)	352.32	52.748	.000	In-service vs. TG-H 74.39 / 127.44	1099.00	.000*	1.02
Main activity	Pre-service (n = 561)	354.20			In-service vs. Pre-service 343.93 / 347.10	36408.50	.870	.012
	TG-H (n = 43)	595.79			Pre-service–TG-H 288.10 / 490.35	3984.00	.000*	.624
Cool down	In-service (n = 39)	56.77	25.369	.000	In-service vs. TG-H 23.38 / 34.50	132.00	.018	.669
	Pre-service (n = 126)	101.17			In-service vs. Pre-service 53.38 / 92.17	1302.00	.000*	.735
	TG-H (n = 12)	66.00			Pre-service–TG-H 72.50 / 38.00	378.00	.004*	.501

## Discussion

The aims of this study were to analyze eTL in tasks designed by in-service and pre-service teachers for teaching handball in primary education and to compare them to the tasks in a teaching unit designed using the tactical games approach (TGA) model. Knowledge of the eTL provoked by the tasks proposed by the teachers in the PE class is necessary to optimize learning, physical condition and foment students´ healthy habits. Moderate to vigorous physical activities are necessary due to the benefits that they provide for health indicators [40]. Adequate training tasks promote favorable physical and physiological adaptations, and reduce the probability of suffering disease and injuries [41]. Teaching methodologies used for sports initiation have a direct relation with eTL [10].

Within the first objective, the eTL planned by the teachers was initially identified. From a qualitative point of view, the eTL in the sessions designed by the in-service and pre-service teachers can be considered low as it did not score more than 18 points on the SIATE scale (6 to 30 points) [16]. These findings are similar to those of studies carried out on the planning of teaching units on team sports by pre-service [21] and in-service teachers [42]. A low eTL was found in the tasks designed for football and basketball using a technical or direct instruction model [19, 20]. It has been shown that teachers plan tasks with a low intensity of external load, failing to cover the demands of intense physical load proposed by the WHO for schoolchildren.

Next, the association of learning situations and means of learning with the eTL of the tasks was analyzed. An association was identified between unopposed learning situations and exercise-based learning media with a low eTL, while tasks with a higher eTL were associated with situations played with opposition. The tasks that follow methodologies based on direct instruction are usually based on exercises [24], with rigid organizations with limited participation, causing a weak response to the load experienced by the students. Exercise-based learning tasks and unopposed situations were associated with low and very low external load in both the warm-up phase and the main part of the physical education session. In situations played with opposition, small games were associated with a high external load and full games with a very high external load [22].

Teachers can manipulate the eTL provoked by the tasks using the modification of their structural and formal parameters, like the rules of the game, the size of the space, the number of players involved, the presence of the goalkeeper, the use of scoring, the duration of the task and/or the encouragement of the teacher. This modification of the elements that define and organize a task provoke different physical, technical and tactical responses in the players [43, 44], which can be taken advantage of to improve the learning and physical fitness of the students. However, it is necessary to collect more information on the effects of the different types of task (Full Game, Small Sided-Game, Exercises,…), for the different age groups in the training stages, both on the effectiveness of the learning of each sport, and the effects on the players’ iTL and eTL and their physiological implications [45, 46].

The second aim was if the teachers in pre-service and in-service plan the load of the tasks to be lower than those of a lesson plan under a tactical games model. The literature has determined that the tasks planned under the tactical games model have higher intensities than those planned under direct instruction [4]. A lesson plan was validated through the tactical games model to compare the tasks designed by the teachers. The tasks designed using the methodology centered on the sport, tactical games model, presented a high eTL, in the range of 19 to 24 points on the SIATE scale [16]. The tasks planned for teaching football and basketball in PE classes using models centered on the student and the understanding of the game present higher eTL [19, 20] and foment greater development of physical aptitudes in the students [10].

Regarding the tasks planned according to the TG-H model there are more cases than expected using a degree of opposition in the categories of numerical equality or with opposition with one more player. This type of tasks provokes higher levels of eTL because of the influence of the competitive load and cognitive implication [22]. However, with respect to the in-service teachers there are more cases than expected of tasks without opposition, which are habitual in planning which follows the direct instruction model [19, 20, 47]. These are application exercises which have a low eTL due to the limited cognitive load [48] and involvement, and the competitive load that they present [22]. The use of this type of activities has been confirmed in the tasks designed for invasion sports by pre-service teachers [22, 49].

The in-service teacher plans the tasks eliciting two responses in the density of the task, either with very low or high intensity. In the pre-service teacher, a high density of tasks predominates, while in the TG-H model the proposed tasks are of high intensity. In the TG-H model the tasks present intensity and rest, due to the repetition of small-sided games which end with a goal or recovery of the ball on the part of the defenders and/or attackers. In the game situations, with the presence of defenders, learning is more contextualized, with more efficient movements and a greater intensity without the need to reach high speeds [10, 50]. Small-sided games increase the participation and involvement of the learners [51], present an increase in accelerations compared with actual play [52], and impose a high physiological demand on the students [53]. In the TG-H lesson plan SSG were manipulated by modifying the constraints, for example the space, the number of players, and the place where the game situations were restarted [30].

In the competitive load involved in the tasks designed by the in-service teachers there was a predominance of “activities without competition” and matches, while the pre-service teachers used “tasks for learning technique” and “tasks with opposition and scoring”. This management of the competitive load denotes a clear preference, in both types of teachers for the technical models based on direct instruction [54]. TG-H planning used the resource of the competitive load to better effect, including more tasks with opposition in which the score counts, as well as matches with all their variants. The tactical model for the teaching of invasion sports uses groups of players where there is rivalry between the participants, permitting internal competition and greater cognitive implication on the part of the participants [54]. All these elements are inherent in the sport itself. The design of tasks in which there is confrontation and competition increases the intensity of the task and the students’ motivation [10]. Furthermore, the selection of tasks which permit simultaneous participation by a large number of students increases the class load.

Cognitive implication is closely related to the grouping of the students. While the in-service teachers more often used situations in which the students have to relate to a small group of companions and opponents, showing mastery of group control, the pre-service teachers used tasks without relating to companions or groups with just one companion or opponent. The TG-H had recourse to relations among small groups of companions and opponents, as well as full game situations. The increase in the number of interactions implies an increase in the load experienced by the student [17]. The design of teaching tasks for handball should favor cognitive implication using the continual relation with opponents and companions, distancing itself from the more traditional methodologies centered on the individual development of technical skills.

In order to increase task load, it is more efficient to use a methodology centered on the game, paying attention to the organization of the task so that there is a high percentage of students participating simultaneously, proposing game situations with opposition thus favoring greater competitive load and cognitive implication, and where there is adequate density in the activity/pause ratio.

It was confirmed that there were differences between the planning of the in-service and pre-service teachers and that carried out using the TG-H model in all the variables except game space. The eTL in the TG-H model was significantly higher, with a high effect size, than in the planning performed by the in-service and pre-service teachers. So, why do teachers not plan using models centered on the game? The teachers in their training phase state that they are lacking the knowledge to plan tasks based on TG [47], needing programs that would serve as models for their application and subsequent development in their professional evolution [55].

The analysis of the eTL as a function of the parts of the session corroborated that in the warm-up and main activity the TG-H model revealed a higher eTL than those designed by the in-service and pre-service teachers, which provoked a low load in the students. The warm-up was of low intensity and the main activity should present higher physical and motor demands with varying intensity. The TG-H model proposed by the experts, through the adequate use of the conditioners that influence the final load imposed on the students, provoked stimuli which favor the development of their physical fitness in a context of specific learning.

As limitations of the study, it should be indicated that the number of sessions of the TU, less than eight, designed by the in-service teachers is low for the learning of a sport. This low number is due to the numerous contents that are developed in an annual sports education program. Pre-service teachers plan with more sessions than in-service teachers.

## Conclusions

Differences have been identified in the variables that define the subjective external load that is supported by the students in the development of the lesson plan proposed by the in-service and pre-service teachers and the group of experts. The teachers should develop their task designs from the pre-service and in-service phases towards models similar to the one planned by the experts, TG-H, using the modification of the constraints that affect the physical response of the students.

The present study showed that in-service and pre-service teachers plan tasks for the learning of handball with a low eTL, both in the whole session and in the phases (warm-up and main activity) where the objectives and contents of the session are developed. A high eTL was evident in the lesson plan designed using a model centered on the game and the understanding of the tactics (tactical games). The designing of tasks with opposition, with all the students participating simultaneously, using SSG and full games, to favor cognitive implication and the competitive load, improves the levels of eTL inherent in the task.

The SIATE instrument can be an effective and inexpensive tool for carrying out a prior analysis of the tasks that the students are going to perform in the PE classes, and assess the eTL, as it permits the teachers to discover the load supported by the students.

The model of a lesson plan based on the tactical games approach is an example that can be used by teachers to improve their task designing, as there are few validated models that serve as examples for new teachers.

## Supporting information

S1 File (PDF)
