# Supplementary material for: External load of the tasks planned by teachers for learning handball
Source: PLoS One. 2022 Apr 5;17(4):e0265745. doi: 10.1371/journal.pone.0265745 (PMC8982867; doi:10.1371/journal.pone.0265745)
Supplement: S1 File — (PDF) [file pone.0265745.s001.pdf]

Diane Schofield
Translator/Interpreter
Associate of the Chartered Institute
of Linguists nº 31814
N.I.F. 50.295.284 B
C/ Tres Picos, 36
28260 Galapagar (Madrid)
SPAIN
Tel: 91. 8583928

18th February 2022

CERTIFICATE OF REVISION

This is to certify that the article entitled:

"EXTERNAL LOAD OF THE TASKS PLANNED BY TEACHERS FOR LEARNING HANDBALL"

was proofread by Diane Schofield, an associate of the Chartered Institute of Linguists, London, with more than 40 years' experience in the translation and proofreading of scientific texts.

Signed: Diane Schofield

Diane Schofield has worked for many years proofreading and translating for the following Universities: the Polytechnic University of Madrid, the University of Castilla La Mancha, the *University* of the Basque Country (UPV/EHU) (Basque - Euskal Herriko Unibertsitatea (EHU), the Distance University (UNED), the University of Extremadura, the Camilo José Cela University, the University of Rennes (France), as well as the Spanish Ministry of Education, the Spanish High Council for Sport, the Royal Spanish Swimming Federation, and many other official Spanish institutions.
